# Inhibition of LXR controls the polarization of human inflammatory macrophages through upregulation of MAFB

**DOI:** 10.1007/s00018-023-04745-4

**Published:** 2023-03-17

**Authors:** Arturo González de la Aleja, Cristina Herrero, Mónica Torres-Torresano, María Teresa Schiaffino, Alejandro del Castillo, Bárbara Alonso, Miguel A. Vega, Amaya Puig-Kröger, Antonio Castrillo, Ángel L. Corbí

**Affiliations:** 1 Myeloid Cell Laboratory, Centro de Investigaciones Biológicas, CSIC, Ramiro de Maeztu 9, 28040 Madrid, Spain; 2 Unidad de Inmuno-Metabolismo e Inflamación, Instituto de Investigación Sanitaria Gregorio Marañón (IiSGM), Hospital General Universitario Gregorio Marañón, Madrid, Spain; 3 Unidad de Biomedicina (Unidad Asociada al CSIC), Instituto Universitario de Investigaciones Biomédicas y Sanitarias (IUIBS), Grupo de Investigación Medio Ambiente y Salud, Universidad de Las Palmas de Gran Canaria, Las Palmas, Spain; 4 Instituto Investigaciones Biomédicas “Alberto Sols” (IIBM), Centro Mixto Consejo Superior de Investigaciones Científicas y Universidad Autónoma de Madrid (CSIC-UAM), Madrid, Spain

**Keywords:** Inflammation, Innate immunity, Macrophage Polarization, Transcriptional profile

## Abstract

Monocyte-derived macrophages contribute to pathogenesis in inflammatory diseases and their effector functions greatly depend on the prevailing extracellular milieu. Whereas M-CSF primes macrophages for acquisition of an anti-inflammatory profile, GM-CSF drives the generation of T cell-stimulatory and pro-inflammatory macrophages. Liver X Receptors (LXRα and LXRβ) are nuclear receptors that control cholesterol metabolism and regulate differentiation of tissue-resident macrophages. Macrophages from rheumatoid arthritis and other inflammatory pathologies exhibit an enriched LXR pathway, and recent reports have shown that LXR activation raises pro-inflammatory effects and impairs the acquisition of the anti-Inflammatory profile of M-CSF-dependent monocyte-derived macrophages (M-MØ). We now report that LXR inhibition prompts the acquisition of an anti-inflammatory gene and functional profile of macrophages generated within a pathological environment (synovial fluid from Rheumatoid Arthritis patients) as well as during the GM-CSF-dependent differentiation of human monocyte-derived macrophages (GM-MØ). Mechanistically, inhibition of LXR results in macrophages with higher expression of the v-Maf Avian Musculoaponeurotic Fibrosarcoma Oncogene Homolog B (MAFB) transcription factor, which governs the macrophage anti-inflammatory profile, as well as over-expression of MAFB-regulated genes. Indeed, gene silencing experiments on human macrophages evidenced that MAFB is required for the LXR inhibitor to enhance the anti-inflammatory nature of human macrophages. As a whole, our results demonstrate that LXR inhibition prompts the acquisition of an anti-inflammatory transcriptional and functional profile of human macrophages in a MAFB-dependent manner, and propose the use of LXR antagonists as potential therapeutic alternatives in macrophage re-programming strategies during inflammatory responses.

**Supplementary Information:**

The online version contains supplementary material available at 10.1007/s00018-023-04745-4.

## Introduction

During inflammation, and depending on their developmental origin, tissue location, and prevailing cytokine environment, macrophages can display pro-inflammatory or anti-inflammatory/resolving effector functions [1–3], whose sequential occurrence allows return to homeostasis, and whose modulation (macrophage re-programming) is considered as a therapeutic strategy for inflammatory disorders [4]. M-CSF and GM-CSF have opposite instructing effects on macrophages during inflammatory responses [5, 6]: M-CSF primes macrophages (M-MØ) for acquisition of an anti-inflammatory/reparative profile, whereas GM-CSF primes macrophages (GM-MØ) for T cell-stimulatory and pro-inflammatory activity. Accordingly, M-MØ and GM-MØ exhibit distinct transcriptional profiles [7, 10, 17–19] that resemble resident and inflammatory macrophages in vivo [19–22]. The homeostatic and reparative profile of M-MØ is determined by MAFB [23, 24], a transcription factor that promotes macrophage differentiation [25], inhibits stemness of monocytes and macrophages [26] and has been recently proposed to shape the transcriptome of pathogenic macrophage subsets in severe COVID-19 [27, 28].

LXRα and LXRβ (coded for by *NR1H3* and *NR1H2*, respectively) are ligand-activated transcription factors involved in cellular cholesterol metabolism and macrophage differentiation and specialization [29–34]. LXR ligands also regulate inflammation in macrophages by antagonizing the induction of inflammation-related genes after activation [30, 35], which has led to the recognition of LXR as anti-inflammatory factors [36, 37]. However, LXR activation also exacerbates inflammatory responses [38–41]: the LXR pathway is upregulated in pro-inflammatory macrophages from rheumatoid arthritis (RA) synovial fluid, where it potentiates cytokine release [42], and is significantly enriched in tumor-associated macrophages from colorectal liver metastasis [43]. In addition, LXRα induces a pro-inflammatory trained immunity state in monocytes [44], regulates trained immunity in macrophages [45], and LXR agonists potentiate glycolysis and increase IL1β mRNA levels in human macrophages [46]. Along this line, high *NR1H3* gene expression marks pro-inflammatory macrophages in diffuse large B-cell lymphoma, where it predicts patient survival [47].

We have recently described that LXR activation with synthetic ligands prompts a pro-inflammatory transcriptional profile during the M-CSF-dependent generation of monocyte-derived M-MØ [48], which suggested LXR inhibition as a potential anti-inflammatory strategy. To directly address this issue, we have now determined the transcriptome of GM-MØ generated in the presence of an LXR inhibitor (GSK2033, GSK-GM-MØ) and revealed that LXR inhibition skews the differentiation of GM-CSF-dependent monocyte-derived GM-MØ towards the acquisition of an anti-inflammatory transcriptional and functional profile. Since LXR inhibition also impairs the monocyte inflammatory differentiation under the influence of synovial fluid from Rheumatoid Arthritis patients, our results support LXR as targets for macrophage re-programming strategies during inflammatory responses.

## Materials and methods

### Generation of human monocyte-derived macrophages in vitro and treatments

Human Peripheral Blood Mononuclear Cells (PBMCs) were isolated from buffy coats from anonymous healthy donors (provided by the Centro de Transfusiones de la Comunidad de Madrid) over a Lymphoprep (Nycomed Pharma) gradient according to standard procedures. Monocytes were purified from PBMC by magnetic cell sorting using anti-CD14 microbeads (Miltenyi Biotec, #130-050-201). Monocytes (> 95% CD14^+^ cells) were cultured at 0.5 × 10^6^ cells/ml in Roswell Park Memorial Institute (RPMI 1640, Gibco, #22400,089) medium supplemented with 10% fetal bovine serum (FBS, Biowest, #S181L) (complete medium) for 7 days in the presence of 1000 U/ml GM-CSF (ImmunoTools, #11343123) or 10 ng/ml M-CSF (ImmunoTools, #11343113) to generate GM-CSF-polarized macrophages (GM-MØ) or M-CSF-polarized macrophages (M-MØ), respectively [23]. Unless indicated otherwise, GM-CSF was added every two days and cells were maintained at 37 °C in a humidified atmosphere with 5% CO_2_ and 21% O_2_. Where indicated, macrophages were treated at different time points with one dose of LXR agonist GW3965 [32] (1 μM, Tocris, #2474), LXR inverse agonist GSK2033 [33] (1 μM, Tocris, #5694) or both, using dimethyl sulfoxide (DMSO) as vehicle. In the dual condition, the inverse agonist was added 1-h prior to agonist treatment. When indicated, Synovial Fluid from Rheumatoid Arthritis patients (RASF) was added to monocytes (0.2:1 in complete medium), and cultures were maintained for 72 h. RASF were kindly provided by the Rheumatology Department at Hospital General Universitario Gregorio Marañón. RASF samples were centrifuged (4000 g, 15 min) to remove cells and particulate material, sterile-filtered, aliquoted, and stored at – 80 °C until use. For macrophage activation, cells were treated with 10 ng/ml E. coli 055:B5 lipopolysaccharide (Ultrapure LPS, Sigma-Aldrich). Human cytokine production was measured in macrophage supernatants using commercial ELISA for TNF (BD Biosciences, #555212), IL1β (#DY201), IL-6 (#DY206), IL-10 (#DY217B), Activin A (#DY338), CCL2 (#DY279), CCL8 (#DY281), and CCL17 (#DY364) (R&D Systems)] according to the procedures supplied by the manufacturers.

### Small interfering ribonucleic acid (siRNA) transfection

Monocyte siRNA transfection was done as described [49]. Briefly, CD14^+^ Monocytes (1 × 10^6^ cells/well in 12-well plates) were transfected with human MAFB-specific smart Pool (siMAFB, 50 nM) (Dharmacon, #M-009018) using Lipofectamine 3000 (1.5 μl per well, ThermoFisher Scientific, #L3000008) and following the manufacturer´s protocol. siGenome non-targeting siRNA Pool#2 (siCtrl, 50 nM) (Dharmacon, #D-001206) was used as negative control siRNA. Six hours after transfection, culture medium was renewed and cells were treated with 1 μM GSK2033 and GM-CSF, and allowed to recover from transfection in complete medium for 3 or 7 days before lysis.

### Quantitative real-time RT-PCR (qRT-PCR)

Total RNA was extracted using the total RNA and protein isolation kit (Macherey–Nagel, #740933.250). RNA samples were reverse-transcribed with High–Capacity cDNA Reverse Transcription reagents kit (Applied Biosystems, #10400745) according to the manufacturer’s protocol. Real-time quantitative PCR was performed with LightCycler® 480 Probes Master (Roche Life Sciences) and Taqman probes on a standard plate in a Light Cycler® 480 instrument (Roche Diagnostics). Gene-specific oligonucleotides were designed using the Universal Probe Library software (Roche Life Sciences). Results were normalized to the expression level of the endogenous references genes *TBP*, *HPRT1* or *GAPDH* and quantified using the ΔΔCT (cycle threshold) method.

### Western blot

GM-MØ cell lysates were subjected to SDS-PAGE (25–50 μg unless indicated otherwise) and transferred onto an Immobilon-P polyvinylidene difluoride membrane (PVDF; Millipore, #IPVH00010). After blocking the unoccupied sites with 5% non-fat milk diluted in Tris-Buffered Saline plus Tween 20 (TBS-T), protein detection was carried out with antibodies against MAFB (Sigma Aldrich, #HPA005653). Protein loading was normalized using an antibody against vinculin (Sigma-Aldrich, #V9131). Quimioluminiscence was detected in a Chemidoc Imaging system (BioRad) using SuperSignal™ West Femto (ThermoFisher Scientific, #34,094).

### Mixed leukocyte reaction (MLR)

Untreated or treated GM-MØ were detached using Trypsin–EDTA (Gibco, #25,200,056) at 37 °C, and re-plated in a 96-well U-bottom ^4^plate in RPMI with 5% human AB serum (Lonza, #4W-820) for 24 h. Allogeneic T lymphocytes were isolated from PBMCs using anti-CD3 magnetic beads (Miltenyi Biotec, #130–050-101), and co-cultured with macrophages (10:1 T lymphocyte:macrophage ratio) for 6 days in RPMI with 5% human AB serum. Then, ^3^H-Thymidine (1 μCi/well, Perkin Elmer, #NET027E001MC) was added and, after 18 h, radioactivity was transferred to a filter and thymidine counts measured in a scintillation counter (Perkin Elmer).

### RNA-sequencing and data analysis

RNA was isolated from three independent GM-MØ samples generated from monocytes exposed to a single dose of DMSO, 1 μM GW3965, 1 μM GSK2033, or both, at the beginning of the 7-day differentiation process. In other set of experiments, RNA was isolated from two preparations of CD14^+^ monocytes exposed to either DMSO (vehicle) or 1 μM GSK2033 for 1 h and then cultured for 3 days in complete medium supplemented with 20% RASF (six independent samples). Sequencing was done on a BGISEQ-500 platform (https://www.bgitechsolutions.com). RNAseq data were deposited in the Gene Expression Omnibus (http://www.ncbi.nlm.nih.gov/geo/) under accession GSE156696 and GSE181313. On average, 88.04 M reads per sample were generated and clean reads were mapped to the reference (UCSC Genome assembly hg38) using Bowtie2 (average mapping ratio to reference genome, 91.82%) [50]. Gene expression levels were calculated by using the RSEM software package [51], and differential gene expression was assessed by using the R-package DESeq2 algorithms using the parameters Fold Change > 2 and adjusted *p* value < 0.05. Heatmaps and clustering were done using the Genesis software (http://genome.tugraz.at/genesisclient/) [52]. Differentially expressed genes were analyzed for annotated gene sets enrichment using ENRICHR (http://amp.pharm.mssm.edu/Enrichr/) [53, 54], and enrichment terms considered significant with a Benjamini-Hochberg-adjusted *p* value < 0.05. For gene set enrichment analysis (GSEA) (http://software.broadinstitute.org/gsea/index.jsp) [55], gene sets available at the website, as well as gene sets generated from publicly available transcriptional studies (https://www.ncbi.nlm.nih.gov/gds), were used.

### Statistical analysis

For comparison of means of three or more groups, statistical significance of the generated data was evaluated using a one-way repeated measures ANOVA test with a post-hoc Tukey test for multiple comparisons between groups. In all cases, *p* < 0.05 was considered as statistically significant.

## Results

### LXR Inhibition reverts the pro-inflammatory effect of Rheumatoid Arthritis synovial fluid (RASF) on human monocytes and prompts the acquisition of a gene profile that resembles anti-inflammatory monocyte-derived macrophages

**Fig. 1:**
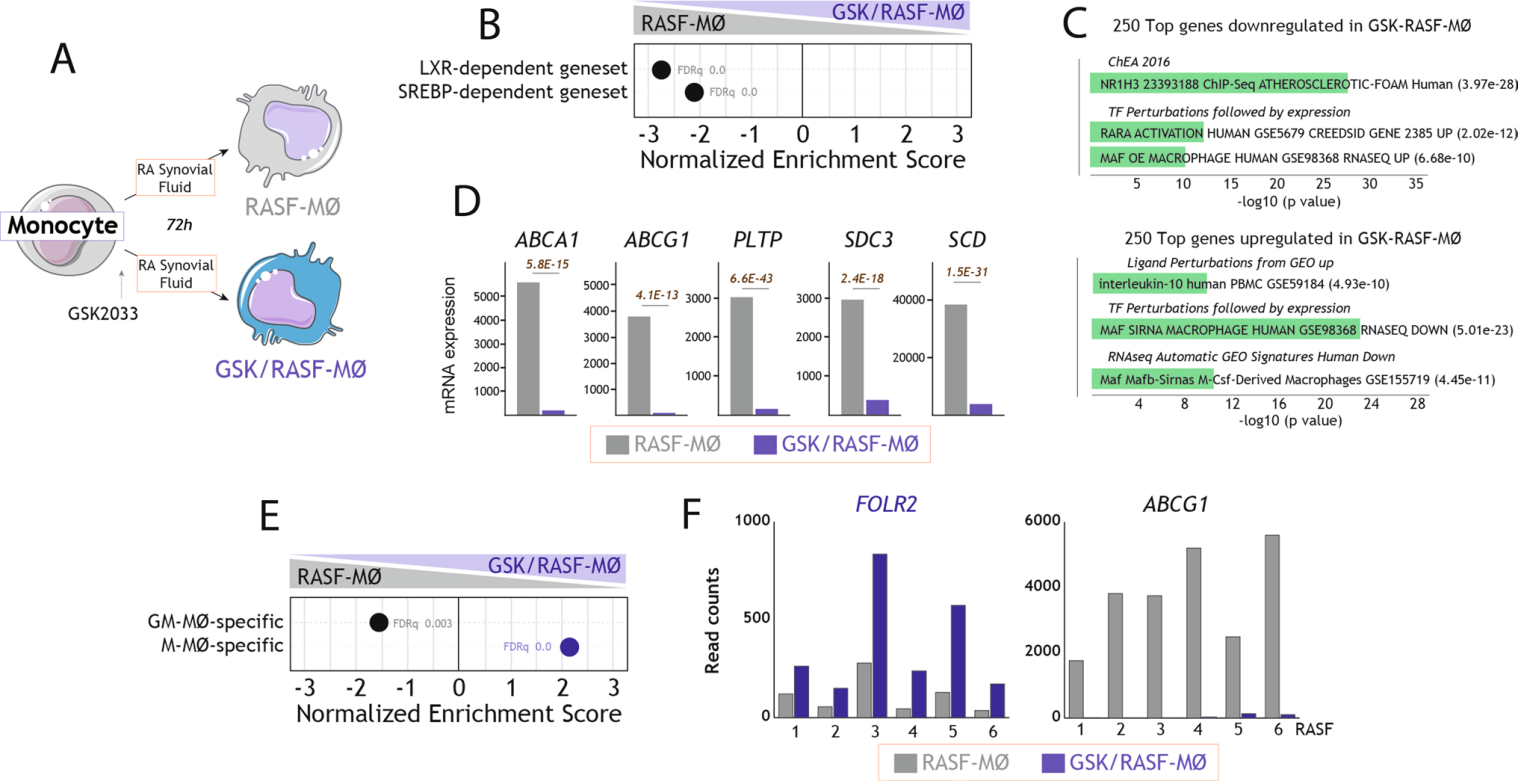
LXR inhibition alters the transcriptome of monocytes differentiated in the presence of Rheumatoid Arthritis Synovial Fluid. **A** Schematic representation for the generation of monocyte-derived macrophages in the presence of Rheumatoid Arthritis Synovial Fluid (RASF), with (GSK/RASF-MØ) or without (RASF-MØ) exposure to GSK2033. **B** GSEA of LXR- and SREBP-dependent gene sets on the ranked comparison of the GSK/RASF-MØ and RASF-MØ transcriptomes. Normalized Enrichment Score (NES) and False Discovery rate q value (FDRq) are indicated. **C** Gene ontology analysis of the Top 250 genes downregulated (upper panel) or upregulated (lower panel) in GSK/RASF-MØ using Enrichr and the indicated databases. **D** mRNA expression (RNAseq Read counts) of the indicated genes in GSK/RASF-MØ and RASF-MØ generated using six independent Rheumatoid Arthritis synovial fluids. **E** GSEA of M-MØ- and GM-MØ-specific gene sets (GSE68061) on the ranked comparison of the GSK/RASF-MØ and RASF-MØ transcriptomes. Normalized Enrichment Score (NES) and False Discovery rate q value (FDRq) are indicated. **F** mRNA expression (RNAseq Read counts) of the indicated genes in GSK/RASF-MØ and RASF-MØ generated using six independent Rheumatoid Arthritis synovial fluids

**Fig. 2:**
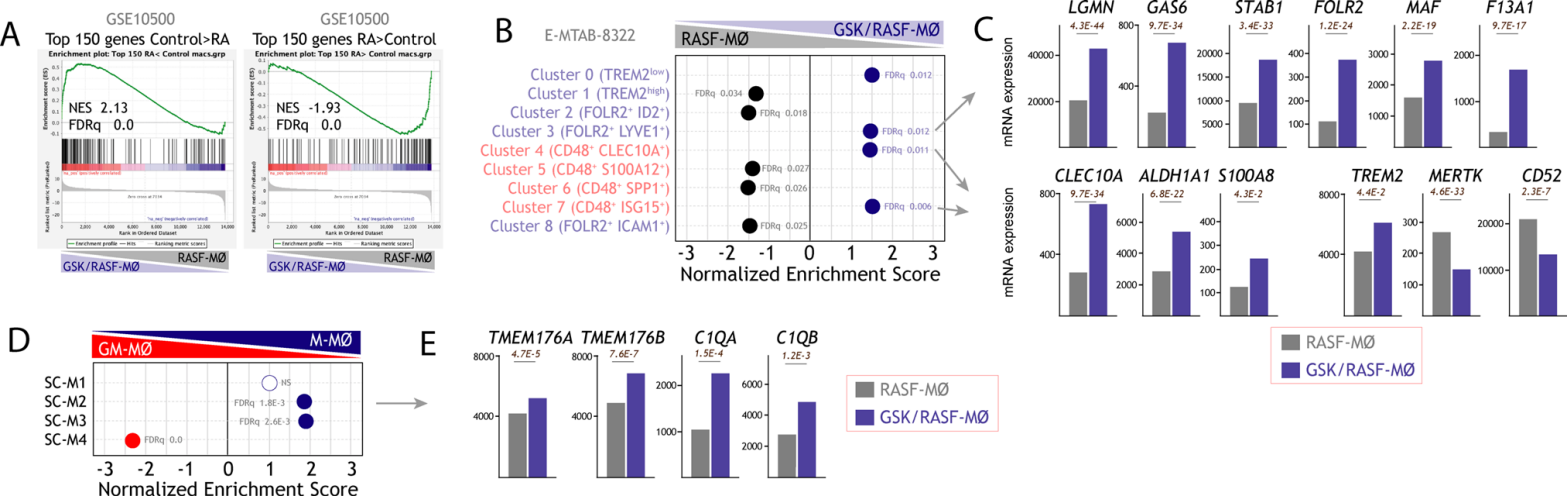
Differential expression of LXR and LXR-regulated genes in GM-MØ and M-MØ. **A** GSEA of genes preferentially expressed by control or RA-specific macrophages (GSE10500) on the ranked comparison of the GSK/RASF-MØ and RASF-MØ transcriptomes. Normalized Enrichment Score (NES) and False Discovery rate q value (FDRq) are indicated. **B** Summary of GSEA of the synovial macrophage gene clusters defined in [61] (https://www.ebi.ac.uk/arrayexpress/experiments/E-MTAB-8322/, E-MTAB-8322) on the ranked comparison of the GSK/RASF-MØ and RASF-MØ transcriptomes. Normalized Enrichment Score (NES) and False Discovery rate q value (FDRq) are indicated. **C** mRNA expression (RNAseq Read counts) of the indicated genes in GSK/RASF-MØ and RASF-MØ generated using six independent Rheumatoid Arthritis synovial fluids. **D** Summary of GSEA of the synovial macrophage gene clusters defined in [62] (ImmPort; https://www.immport.org/shared/study/SDY998, SDY998) on the ranked comparison of the anti-inflammatory M-MØ and pro-inflammatory GM-MØ transcriptomes (GSE68061). Normalized Enrichment Score (NES) and False Discovery rate q value (FDRq) are indicated. **E** mRNA expression (RNAseq Read counts) of the indicated genes in GSK/RASF-MØ and RASF-MØ generated using six independent Rheumatoid Arthritis synovial fluids

We have previously demonstrated that LXR activation promotes the acquisition of an inflammatory transcriptional and functional profile in human monocyte-derived macrophages [48]. Since synovial LXR expression varies among RA pathotypes (https://peac.hpc.qmul.ac.uk) [56] (Supplementary Fig. 1A) and LXR target gene expression is upregulated in pro-inflammatory macrophages from the synovial fluid of Rheumatoid Arthritis patients (RASF-MØ) [39, 42], we proposed that LXR inhibition might tone down macrophage inflammatory ability. To assess this hypothesis, we evaluated whether inhibition of LXR activity modifies the macrophage-polarizing ability of the Rheumatoid Arthritis synovial fluid (RASF). To that end, monocytes were exposed for 72 h to RASF in the presence or absence of the LXR inverse agonist GSK2033 (1 μM) [33] (Fig. 1A). The transcriptome of the resulting macrophages (RASF-MØ, GSK-RASF-MØ) evidenced that LXR inhibition alters the expression of more than 2000 genes (Supplementary Fig. 1B). As expected, GSK-RASF-MØ had a significantly lower expression of genes regulated by either LXR or SREBP [57] (Fig. 1B, C), including *bona fide* LXR target genes like *ABCA1* and *ABCG1* (Fig. 1D). Besides, the gene profile of GSK-RASF-MØ was enriched in MAF- and MAFB-dependent genes, as well as in IL-10-regulated genes (Fig. 1C), and showed a strong over-representation of anti-inflammatory M-MØ-specific genes [7, 10, 17–22] (GSE68061) (Fig. 1E, F, Supplementary Fig. 1C). As a representative example, GSK-RASF-MØ had an augmented expression of *FOLR2* (Fig. 1F), which characterizes tissue-resident macrophages from numerous organs [58–60]. These results indicate that LXR inhibition opposes the pro-inflammatory effect of Rheumatoid Arthritis synovial fluid while enhances the expression of genes that mark anti-inflammatory (M-CSF-dependent) macrophages. Indeed, the GSK-RASF-MØ transcriptome showed a significant reduction of the genes that globally characterize macrophages from the synovial fluid of RA patients (GSE10500) (Fig. 2A, Supplementary Fig. 1D), and augmented expression of the genes that identify synovial macrophages clusters associated to repair responses and disease remission in the synovium of RA patients [61] (e.g., *TREM2*, *GAS6*) (https://www.ebi.ac.uk/arrayexpress/experiments/E-MTAB-8322/, E-MTAB-8322) (Fig. 2B, C), as well as synovial macrophage clusters that perform homeostatic needs (SM-C2, SM-C3) (ImmPort; https://www.immport.org/shared/study/SDY998, SDY998) [62] like *C1QA* and *C1QB* (Fig. 2D, E, and Supplementary Fig. 1E). Therefore, LXR inhibition impairs the acquisition of the inflammatory profile of macrophages from Rheumatoid Arthritis synovium and influences the expression of genes that mark specific populations of macrophages within human synovium.

### LXR inhibition shifts the GM-CSF-dependent differentiation of GM-MØ towards the anti-inflammatory side

**Fig. 3:**
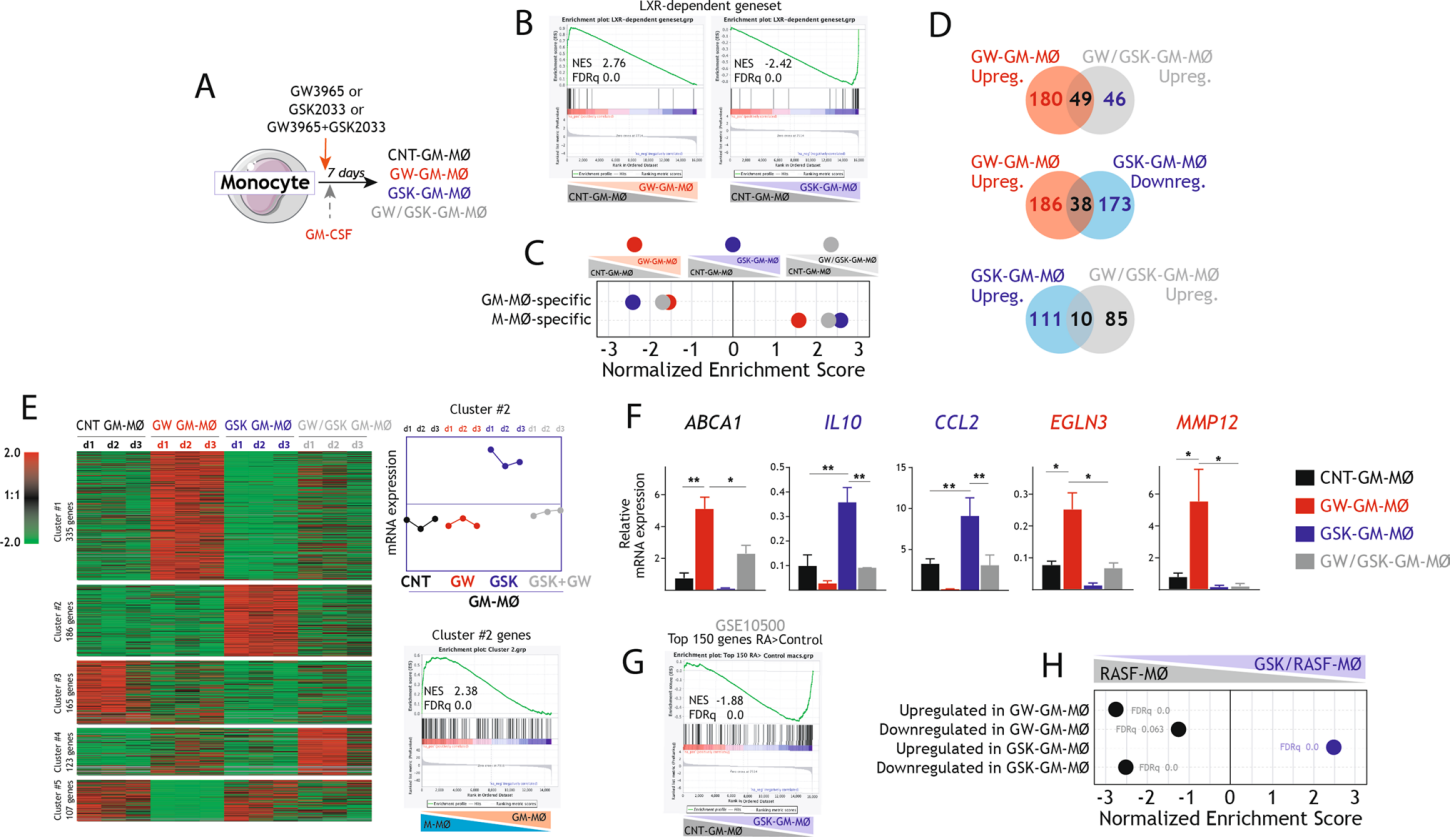
Effect of LXR modulation on the generation of monocyte-derived pro-inflammatory GM-MØ.** A** In vitro generation of control GM-MØ (CNT-GM-MØ), GW3965-GM-MØ (GW-GM-MØ), GSK2033-GM-MØ (GSK-GM-MØ) and GSK2033 + GW3965-GM-MØ (GW/GSK-GM-MØ) before RNA isolation and RNA-sequencing. Control GM-MØ were exposed to DMSO in parallel. **B** GSEA of LXR-dependent genes on the ranked comparison GW-GM-MØ vs. CNT-GM-MØ and GSK-GM-MØ vs. CNT-GM-MØ. Normalized Enrichment Score (NES) and False Discovery rate q value (FDRq) are indicated. **C** GSEA of the M-MØ-specific and GM-MØ-specific gene sets (GSE68061) on the ranked comparisons of the indicated transcriptomes. **D** Comparison of genes differentially expressed in the indicated macrophage types. **E** (*Left panel*) Unsupervised clustering of differentially expressed genes (|log_2_FC|> 1 and adjp < 0.05) between CNT-GM-MØ and the transcriptomes of GM-MØ generated in the presence of GW3965, GSK2033 or both. For each gene, mRNA expression level in the three donors is represented after normalizing gene expression and k-means clustering using Genesis (http://genome.tugraz.at/genesisclient/). (*Upper right panel*) Average expression level of the Cluster #2 genes in CNT-GM-MØ, GW-GM-MØ, GSK-GM-MØ and GW/GSK-GM-MØ. (*Lower right panel*) GSEA of the Cluster #2 genes on the ranked comparison of the M-MØ and GM-MØ transcriptomes (GSE68061). **F** Relative expression of the indicated LXR-dependent genes in CNT-GM-MØ, GW-GM-MØ, GSK-GM-MØ and GW/GSK-GM-MØ. Mean ± SEM of three independent experiments is shown (*p < 0.05, ** *p* < 0.01). **G**. GSEA of the genes preferentially expressed by RA-specific macrophages (GSE10500) on the ranked comparison of the GSK-GM-MØ and CNT-GM-MØ transcriptomes. Normalized Enrichment Score (NES) and False Discovery rate q value (FDRq) are indicated. **H** Summary of GSEA of the genes upregulated or downregulated in GW-GM-MØ or GSK-GM-MØ on the ranked comparison of the GSK/RASF-MØ and RASF-MØ transcriptomes. Normalized Enrichment Score (NES) and False Discovery rate q value (FDRq) are indicated

GM-CSF levels are increased in the serum, synovial fluid and bone marrow of patients with RA, especially at the chronic stage [63], and its relevance in RA disease development is supported by the encouraging results yielded by clinical trials targeting GM-CSF [64]. Since macrophages from the synovium of patients with active rheumatoid arthritis (GSE10500) [65] exhibit a pro-inflammatory gene profile that resembles that of GM-CSF-dependent pro-inflammatory monocyte-derived macrophages (GM-MØ) [21] (Supplementary Fig. 2A), we next sought to determine whether LXR inhibition affected the acquisition of the pro-inflammatory profile of GM-CSF-dependent macrophages. To that end, monocytes were exposed to either 1 μM GSK2033 (LXR inverse agonist), 1 μM GW3965 (LXR agonist), or both, at the beginning of the differentiation process with GM-CSF (Fig. 3A). RNAseq on the resulting macrophages (CNT-GM-MØ, GW-GM-MØ, GSK-GM-MØ and GW/GSK-GM-MØ) showed that LXR ligands greatly altered the acquisition of the transcriptome of GM-MØ (Supplementary Fig. 2B) including, as expected, the expression of LXR target genes (Fig. 3B). More importantly, GSK-GM-MØ exhibited a strong over-representation of the M-MØ-specific "Anti-inflammatory gene set" and a negative enrichment of the GM-MØ-specific "Pro-inflammatory gene set" (Fig. 3C, Supplementary Fig. 2C), in agreement with the ability of GSK2033 to enhance the expression of M-MØ-specific genes in RASF-MØ (Fig. 1E). Comparison of the genes differentially expressed by GW-GM-MØ, GSK-GM-MØ and GW/GSK-GM-MØ (relative to CNT-GM-MØ) not only revealed that GSK2033 impairs the effect of GW3965 (Fig. 3D–F, Supplementary Fig. 2C,D) but evidenced the existence of a cluster of genes (designated Cluster#2) whose expression is specifically augmented in GSK-GM-MØ (Fig. 3E) as well as in anti-inflammatory M-MØ (Supplementary Fig. 2E) and GSK/RASF-MØ (Supplementary Fig. 2F). Analysis of a validation set of samples confirmed that GSK-GM-MØ express higher levels of M-MØ-specific genes like *IL10* and *CCL2*, and that GW/GSK-GM-MØ had lower levels of the GM-MØ-specific genes *EGLN3* and *MMP12* than GW-GM-MØ (Fig. 3F). GSK-GM-MØ showed an over-representation of genes that distinguish control from RA synovial macrophages (GSE10500) [65] (Fig. 3G), and the genes upregulated in GSK-GM-MØ greatly coincided with those upregulated in GSK/RASF-MØ (Fig. 3H). Therefore, all these results confirm that LXR inhibition impairs the acquisition of the inflammatory gene profile that characterizes GM-CSF-dependent monocyte-derived macrophages and promotes the expression of genes that define M-CSF-dependent monocyte-derived anti-inflammatory macrophages. Of note, analysis of a validation set of samples showed that the transcriptional consequences of LXR inhibition were stronger at the start of the monocyte-to-GM-MØ differentiation process (Supplementary Fig. 3A, B) and were observed with various GM-CSF exposure and dose regimens (Supplementary Fig. 3C,D).

### LXR inhibition limits macrophage pro-inflammatory and immuno-stimulatory functions

**Fig. 4:**
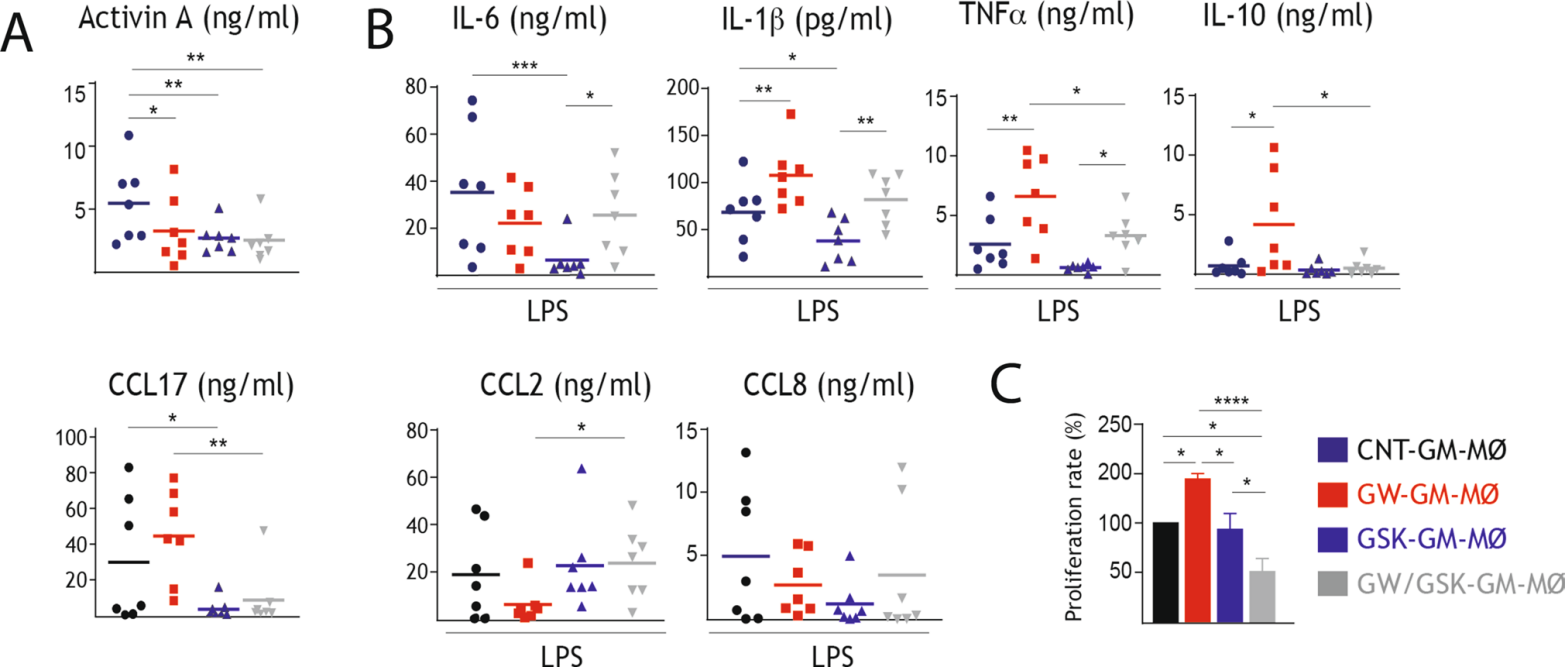
Functional consequences of LXR inhibition during generation of pro-inflammatory M-MØ.** A** Production of activin A and CCL17 in CNT-GM-MØ, GW-GM-MØ, GSK-GM-MØ and GW/GSK-GM-MØ, as determined by ELISA. **B** Production of the indicated cytokines and chemokines in LPS-treated (10 ng/ml, 18 h) CNT-GM-MØ, GW-GM-MØ, GSK-GM-MØ and GW/GSK-GM-MØ, as determined by ELISA. In **A**, **B**, mean ± SEM of six-eight independent samples are shown (*p < 0.05; **p < 0.01; ***p < 0.001). **C** T cell-stimulatory capacity (% Proliferation rate) of GM-MØ generated in the presence of GW3965 (1 μM), GSK2033 (1 μM) or both, as determined in Mixed Leukocyte Reactions (GM-MØ vs. allogeneic CD3^+^ T lymphocyte), using CNT-GM-MØ generated in the presence of DMSO as a control. Mean ± SEM of ^3^H-thymidine incorporation from six independent donors is shown (*p < 0.05; **p < 0.01; ***p < 0.001; ****p < 0.001)

Next, we assessed the functional significance of the transcriptional changes observed upon LXR inhibition. Comparison of the cytokine profile of CNT-GM-MØ, GW-GM-MØ and GSK-GM-MØ in resting conditions indicated that GSK-GM-MØ produced significantly lower levels of activin A and CCL17 (Fig. 4A), whose expression is a characteristic property of GM-MØ. Notably, GSK-GM-MØ also produced significantly lower levels of IL- 6 and IL-1β upon activation with LPS, thus supporting its lower inflammatory nature (Fig. 4B), whereas no significant effect was seen in the LPS-induced production of IL-10 and CCL2 (Fig. 4B). While comparison of the LPS-induced production of TNF and CCL8 from GSK-GM-MØ and CNT-GM-MØ did not reach statistical significance, it is worth noting that GW/GSK-GM-MØ produced significantly less TNF than GW-GM-MØ, further supporting the anti-inflammatory effect of GSK2033 (Fig. 4B). The lower production of pro-inflammatory cytokines from activated GSK-GM-MØ, whose lower expression of *ABCA1* and *ABCG1* might lead to higher intracellular cholesterol levels, agrees with the diminished expression of *TNF*, *IL1B* and *CXCL8* from LPS-stimulated cholesterol-loaded GM-MØ [66]. On the other hand, analysis of the allogeneic T lymphocyte-activating effect did not reveal an alteration of the T-cell stimulatory capacity of GSK-GM-MØ (Fig. 4C) albeit GW/GSK-GM-MØ exhibited a weaker stimulatory capacity than GW-GM-MØ (Fig. 4C, Supplementary Fig. 3E). These results indicate that inhibition of LXR during GM-CSF-driven macrophage differentiation leads to diminished production of pro-inflammatory cytokines and might also affect the acquisition of the macrophage immune-stimulatory capacity. Therefore, LXR inhibition shifts monocytes towards the generation of macrophages with a weaker pro-inflammatory and cytokine profile.

### Molecular mechanisms underlying the macrophage programming effect of LXR inhibition: role of MAFB

**Fig. 5:**
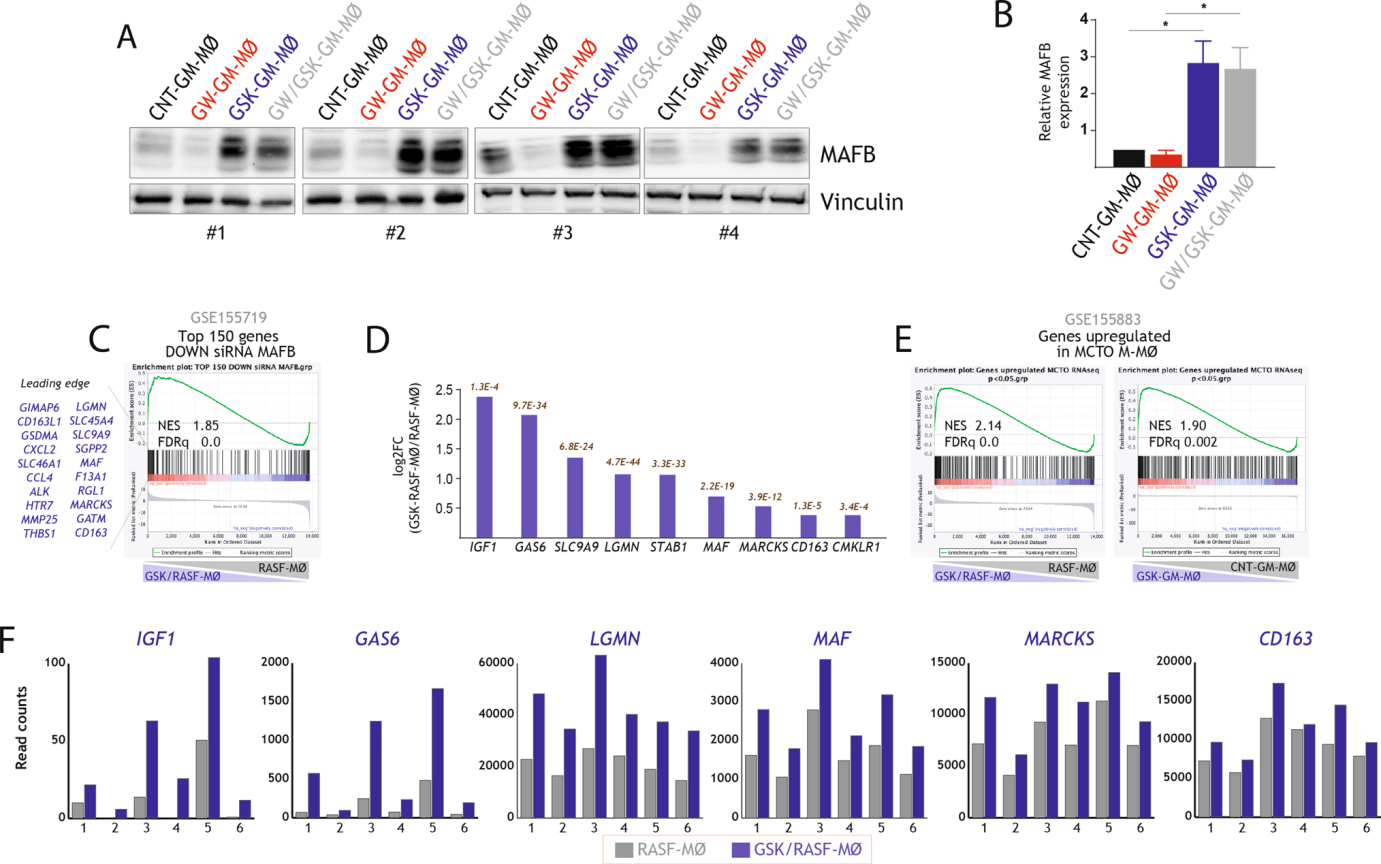
Molecular mechanisms underlying the macrophage-polarizing effect of LXR inhibition. **A** MAFB protein levels in four independent preparations of CNT-GM-MØ, GW-GM-MØ, GSK-GM-MØ and GW/GSK-GM-MØ, as determined by Western blot. For protein loading control purpose, vinculin protein levels were determined in parallel. **B** Quantification of the Western blots shown in A. Mean ± SEM of the relative MAFB protein levels in the four macrophage subtypes from four independent donors are shown (*p < 0.05). **C** GSEA of genes downregulated by siRNA MAFB (GSE155719) on the ranked comparison of the transcriptomes of GSK/RASF-MØ and RASF-MØ transcriptomes. Normalized Enrichment Score (NES) and False Discovery rate q value (FDRq) are indicated. **D** Relative expression of representative MAFB-dependent genes in GSK/RASF-MØ and RASF-MØ. **E** GSEA of the genes upregulated in MCTO M-MØ (GSE155883) on the ranked comparison of the transcriptomes of GSK/RASF-MØ and RASF-MØ transcriptomes (left panel) and GSK-GM-MØ and CNT-GM-MØ transcriptomes (right panel). Normalized Enrichment Score (NES) and False Discovery rate q value (FDRq) are indicated. **F** mRNA expression (RNAseq Read counts) of the indicated MAFB-dependent genes in GSK/RASF-MØ and RASF-MØ generated using six independent Rheumatoid Arthritis synovial fluids

Gene ontology analysis on the transcriptome of GSK/RASF-MØ indicated an over-representation of genes regulated by MAF and/or MAFB (Fig. 1D), which are master regulators for the differentiation of anti-inflammatory M-MØ [23, 24, 67], and a similar enrichment was observed in the GSK-GM-MØ gene profile (“Maf Mafb-Sirnas M-Csf-Derived Macrophages GSE155719” gene set, adj p, 1.160e^−7^). Thus, we analyzed the abundance of MAFB in four independent macrophage preparations. As shown in Fig. 5A, B, MAFB protein expression was much higher in GSK-GM-MØ and GW/GSK-GM-MØ, indicating that LXR inhibition during GM-MØ differentiation increases MAFB. Similarly, and although to a lower extent, MAF protein levels were also higher in GSK-GM-MØ (data no shown). In agreement with the higher expression of MAFB, the gene expression profile of GSK/RASF-MØ was significantly enriched in MAFB-dependent genes (GSE155719) (Fig. 5C, D), as well as genes upregulated in macrophages from a patient with Multicentric Carpo–Tarsal Osteolysis (MCTO, Online Mendelian Inheritance in Man #166300), a very rare autosomal dominant disorder caused by mutations within the MAFB transcriptional activation domain [68, 69] that result in higher MAFB protein levels [23] (Fig. 5E). In fact, and as shown in Fig. 5F, the expression of these MAFB-dependent genes was higher in the six independent GSK/RASF-MØ samples analyzed. Therefore, exposure to an LXR inhibitor like GSK2033 results in enhanced expression of MAFB and MAFB-regulated genes in the context of either RASF or GM-CSF. These results fully agree with the predictions of gene ontology analysis and demonstrate that the anti-inflammatory outcome of LXR inhibition correlates with an enhanced expression of factors that shape the transcriptional and functional profile of anti-inflammatory M-MØ.

**Fig. 6:**
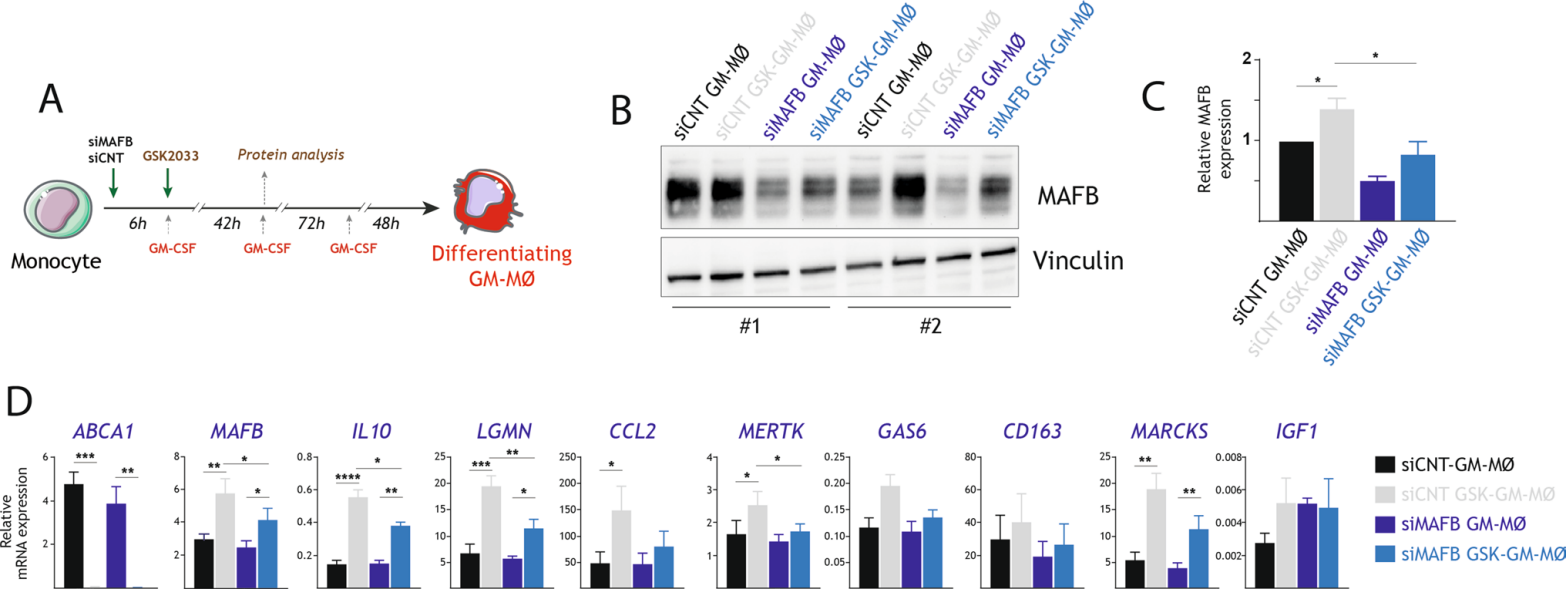
MAFB mediates the transcriptional effect of GSK2033 along monocyte-to-macrophage differentiation.** A** Schematic representation of the generation of GM-MØ in the presence (or absence) of GSK2033 and with or without a previous siRNA-mediated knock-down of MAFB (siMAFB). **B** MAFB protein levels in two independent preparations of differentiating GM-MØ (day 3) generated from monocytes transfected with either siCNT (siCNT GM-MØ) or MAFB-specific siRNA (siMAFB GM-MØ) and subsequently left untreated or exposed to GSK2033 (siCNT GSK-GM-MØ or siMAFB GSK-GM-MØ), as determined by Western blot. For protein loading control purpose, vinculin protein levels were determined in parallel. **C** Quantification of MAFB expression in siCNT GM-MØ, siMAFB GM-MØ, siCNT GSK-GM-MØ and siMAFB GSK-GM-MØ (day 3), as determined by Western blot. Mean ± SEM of the relative MAFB protein levels in the macrophage subtypes from four independent donors are shown (*p < 0.05). **D** Relative mRNA expression of the indicated MAFB-dependent genes in siCNT GM-MØ, siMAFB GM-MØ, siCNT GSK-GM-MØ and siMAFB GSK-GM-MØ at the end of the differentiation protocol (day 7). *ABCA1* expression was evaluated as a readout for LXR activation. Mean ± SEM of three independent experiments are shown (*p < 0.05; **p < 0.01; ***p < 0.001; ****p < 0.0001)

Having demonstrated the link between LXR inhibition and enhanced MAFB protein levels, we next determined whether MAFB mediates the re-programming activity of LXR inhibition. To that end, the effect of 1 μM GSK2033 on GM-MØ differentiation was assessed upon siRNA-mediated silencing of MAFB (Fig. 6A). In this experimental set-up, 1 μM GSK2033 increased the expression of *MAFB* and the GSK2033-mediated upregulation of MAFB was prevented after MAFB silencing in monocytes (Fig. 6B, C). In addition, knocked-down of MAFB significantly reduced the GSK2033-mediated upregulation of *IL10*, *LGMN* and *MERTK*, while the reduction did not reach statistical significance in the case of *CCL2*, *CD163*, *MARCKS*, and *GAS6* (Fig. 6D). Conversely, reduction of MAFB levels did not significantly modify the ability of GSK2033 to blunt *ABCA1* expression (Fig. 6D). As a whole, these results demonstrate that the effect of the LXR inverse agonist GSK2033 on the transcriptome of human monocyte-derived macrophages is mediated, at least partly, through the augmented expression of MAFB.

## Discussion

LXR nuclear receptors are lipid sensors that control crucial aspects of sterol homeostasis and regulate the expression of inflammatory mediators [reviewed in [70]]. Numerous studies have shown, mostly in mouse models, that LXR synthetic ligands suppress inflammation when administered prior to a pathogenic stimuli [70]. However, several recent reports using human cellular models have shown a link between LXR activation and enhanced inflammatory responses, and illustrated the ability of LXR to promote macrophage pro-inflammatory re-programming [35, 38–48]. In the present manuscript, we provide evidences that LXR inhibition favors the acquisition of an anti-inflammatory profile in human monocyte-derived macrophages generated in the presence of GM-CSF or in the context of synovial fluid from Rheumatoid Arthritis patients. The latter is especially relevant because an enhanced activity of LXR has been shown in Rheumatoid Arthritis [39, 40, 42], what supports the physiological relevance of the association between LXR and a pro-inflammatory state in human macrophages and the potential therapeutic value of macrophage re-programming through modulation of LXR. Of note, we have observed that the effects of LXR inhibition are directly related to, and partly mediated by, increased expression of MAFB, a transcription factor that determines the acquisition of an anti-inflammatory, pro-fibrotic and pro-tumoral state in human monocyte-derived macrophages [23, 24, 27, 67, 71, 72]. Our findings are reinforced by studies showing that LXR inhibition impedes the effects of different inflammatory stimuli on macrophage function, as it blocks the upregulation of glycolytic genes in macrophages incubated with plaque homogenates [73] and the ox-LDL-dependent trained immunity in monocytes [45]. Indeed, similar effects are observed with a distinct LXR inverse agonist (SR9238) [[45] and not shown]. Thus, and to the best of our knowledge, our results constitute the first link between LXR inactivation and promotion of a macrophage anti-inflammatory polarization.

Like the pro-inflammatory effect of the LXR agonist on human monocyte-to-macrophage differentiation [48], the re-programming action of LXR inhibition is also cell-specific and differs among monocytes, differentiating and fully differentiated GM-MØ. The fact that LXR inhibition has more profound effects at the monocyte stage (Supplementary Fig. 3B) suggests that LXR factors condition the monocyte to differentiating factors like M-CSF and GM-CSF, and that LXR activity critically determines the levels/activity of factors that drive the differentiation towards M-MØ (MAF, MAFB) or GM-MØ (e.g., activin A). Indeed, we have shown that LXR inhibition affects the expression of MAF/MAFB as well as activin A at the protein level (Figs. 4,5). Interestingly, functional LXR-binding sites have been identified within GM-MØ-specific genes that are upregulated by GW3965 (http://cistrome.org/db/#/; CistromeDB: 69,799) [74]. On the other hand, and although the influence of LXR on MAFB can be direct or indirect, the presence of functional LXRα-binding elements within the human MAFB gene in HT29 colorectal adenocarcinoma (http://cistrome.org/db/#/, CistromeDB: 69,799) [74] suggests that LXR factors might have a direct effect on the expression of genes directly controlling the inflammatory profile of monocyte-derived macrophages. The identification of LXR-regulated genes in M-MØ and GM-MØ by ChIP-Seq should provide a definitive answer to this question. If this postulate holds true in the case of human macrophages, modulation of LXR would constitute a tool for re-programming myeloid cells via control of the expression of regulators of differentiation and polarization.

Interestingly, our analysis of RASF-MØ revealed an upregulation of TREM2, whose expression has been associated with a subset of macrophages with a proangiogenic and immunosuppressive profile in Hepatocellular Carcinoma patients [75]. In the tumoral context, the presence of TREM2^+^ macrophages correlates with a worse prognosis. However, in the context of RA, macrophages with this functional profile might be beneficial to contain the immune over-activation and inflammation of the joints, thus supporting our findings that LXR inhibition skews macrophages to acquire a more anti-inflammatory phenotype.

The partial inhibition of the GSK2033 re-programming effect upon MAFB knockdown might reflect that complete MAFB silencing is required to completely abolish the consequences of LXR inhibition. Alternatively, it is conceivable that other transcriptional programs can be modulated by LXR and thus contribute to the macrophage re-programming action of LXR inhibition. In this regard, and besides the known trans-repression action of LXR on NFκB and AP-1 [76], main drivers of inflammatory macrophage polarization [77], GSK2033 might be altering the LXR ability to promote the interaction between PU.1 and IRF8 [78], two factors that affect macrophage fate and polarization [79, 80]. In addition, LXR activity directly and indirectly affects the activity of SREBP1/2 transcription factors [57], whose contribution to anti- [81] or pro-inflammatory gene expression [82] may have profound effects during monocyte differentiation and macrophage activation. Therefore, in addition to the results presented here on MAFB, LXR inhibition by GSK2033 might exert further influence on pathways that are important for macrophage differentiation and activation, including IRF8 and SREBP1/2, a hypothesis that deserves further attention.

In summary, we describe the feasibility of altering the GM-CSF-driven monocyte-to-macrophage differentiation through inhibition of LXR, which impairs the generation of pro-inflammatory GM-MØ and stimulates the acquisition of an anti-inflammatory transcriptional and functional profile. Taken together with the reverse effects reported for LXR activators [48], these results indicate that modulation of LXR might be a target for macrophage re-programming strategies in pathological conditions. Indeed, pharmacological efforts towards the design LXR isoform-specific, or tissue-restricted, agonists have been conducted in recent years and successfully tested in pre-clinical models of lipid disorders or cancer [83–86]. Thus, the design of LXR modulators for macrophage re-programming purposes in inflammatory diseases would be feasible, selecting those ligands with ability to modify the macrophage inflammatory state without compromising cholesterol metabolism. In addition, our results suggest that LXR-dependent macrophage genes might be useful prognostic/therapeutic markers for human inflammatory diseases, a property that has already been demonstrated for *NR1H3* in the case of diffuse large B-cell lymphoma [47].

### Supplementary Information

Below is the link to the electronic supplementary material.

Supplementary file1 (PDF 427 kb)

## Data Availability

Datasets generated during the current study are available in the Gene Expression Omnibus (http://www.ncbi.nlm.nih.gov/geo/) under accession GSE156696 and GSE181313.
