# Supplementary figures and images for: Inhibition of LXR controls the polarization of human inflammatory macrophages through upregulation of MAFB
Source: Cell Mol Life Sci. 2023 Mar 17;80(4):96. doi: 10.1007/s00018-023-04745-4 (PMC10020776; doi:10.1007/s00018-023-04745-4)

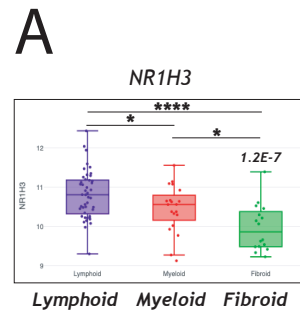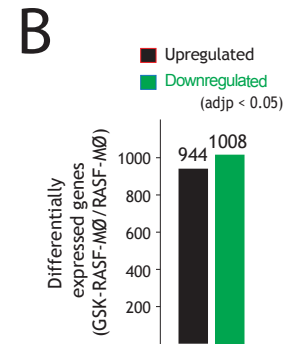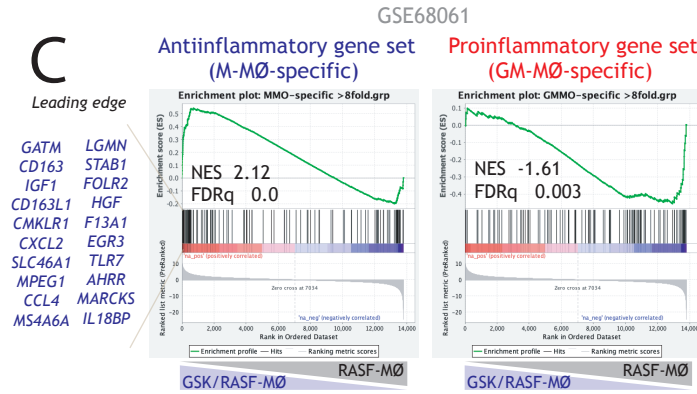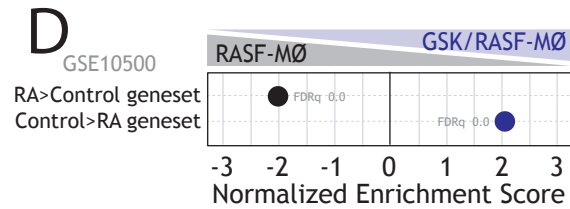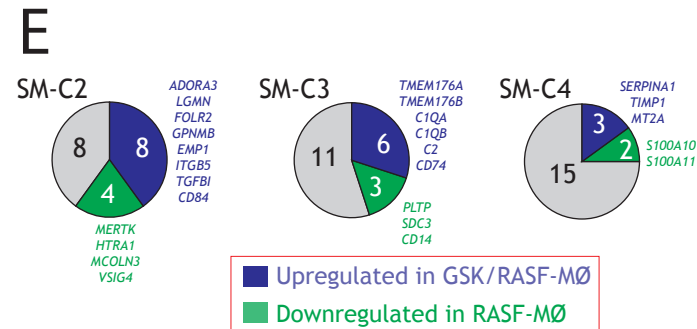

Supplementary Figure 1

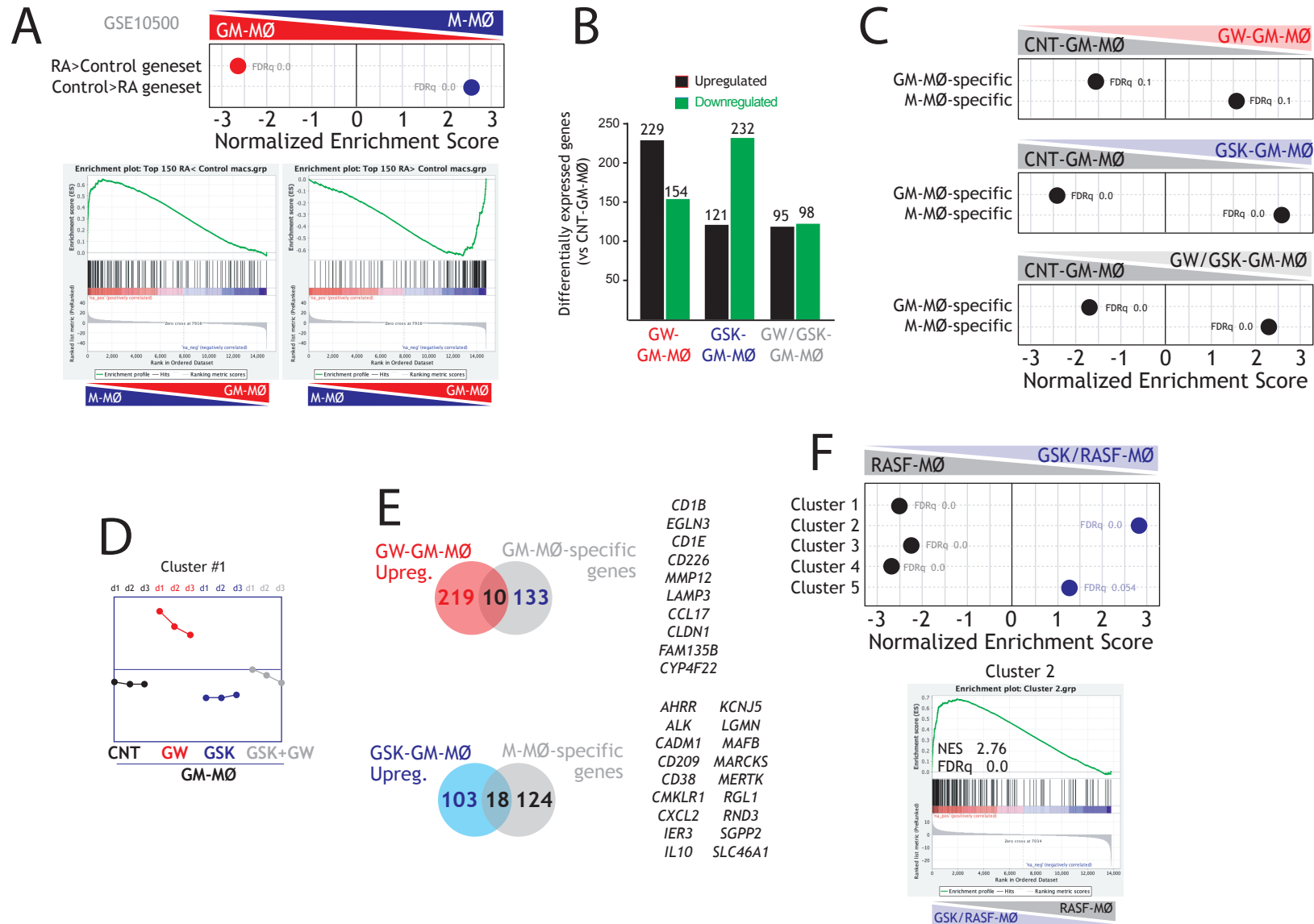

Supplementary Figure 2

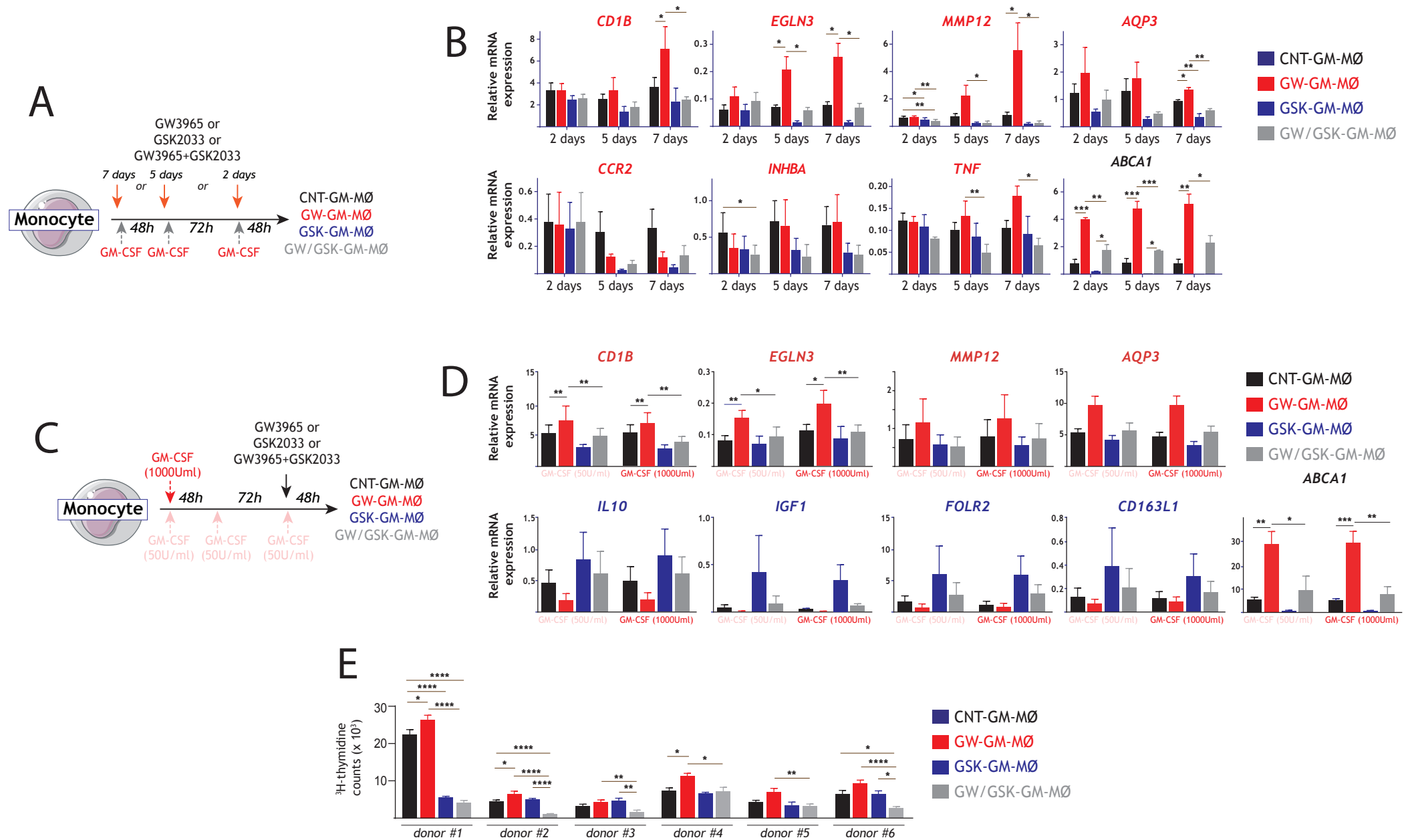

Supplementary Figure 3

Supplement: Supplementary file 1 — Supplementary file1 (PDF 427 kb) [file 18_2023_4745_MOESM1_ESM.pdf]
